# Respiratory characteristics and related intraoperative ventilatory management for patients with COVID-19 pneumonia

**DOI:** 10.1007/s00540-020-02845-0

**Published:** 2020-08-31

**Authors:** Hiroko Aoyama, Kanji Uchida

**Affiliations:** Department of Anesthesiology, Graduate School of Medicine, The University of Tokyo, 7-3-1, Hongo, Bunkyo-ku, Tokyo, 113-8655 Japan

**Keywords:** SARS-CoV-2, COVID-19, Respiratory characteristics, Intraoperative management, Phenotype

## Abstract

A substantial proportion of patients with coronavirus disease 19 (COVID-19) develop severe respiratory failure. Although the exact pathophysiology of severe COVID-19 pneumonia remains unknown and the characteristics of these patients are heterogeneous, the acute respiratory failure often fulfills criteria for acute respiratory distress syndrome (ARDS) and the clinical characteristics are also consistent with what is previously known about ARDS. Cohort studies also report distinctively high association between perioperative COVID-19 and postoperative mortality. In this special article, we review several publications on the pathophysiology of COVID-19, and discuss intraoperative ventilatory management for patients with COVID-19 based on the respiratory characteristics of COVID-19 pneumonia in light of the ongoing controversy of clinical phenotypes.

## Introduction

The current COVID-19 pandemic has caused unprecedented economic burden and stress on both global healthcare systems and in-hospital management of patients infected with severe acute respiratory syndrome coronavirus 2 (SARS-CoV-2). To date we have experienced tremendous advances in our understanding of COVID-19. However, there remains much to be learned about how SARS-CoV-2 affects the lungs to cause lasting lung injury and about how we should approach treatment of these patients. There is currently no treatment specifically approved for COVID-19. Refractory hypoxia due to COVID-19 pneumonia is the primary cause of death among COVID-19 associated patients [1, 2] though relatively little is known about respiratory characteristics of COVID-19 pneumonia. Furthermore, for anesthesiologists who take care of patients undergoing surgery with possible COVID-19 infection, the association between intraoperative anesthetic management, including respiratory management, and postoperative outcomes is a key question that remains unclear. The goal of the current review is to describe postoperative outcomes for patients who were perioperatively confirmed with COVID-19 and to further the discussion related to respiratory characteristics of COVID-19 pneumonia. We also propose intraoperative ventilation strategies for anesthesia care-providers who treat patients scheduled for surgery under possible COVID-19 infection.

### COVID-19 associated postoperative outcomes

The relevant literature on intraoperative ventilatory management in COVID-19 patients is scarce. A case–control cohort that evaluated postoperative outcome of patients who perioperatively confirmed positive for polymerase chain reaction (PCR) of SARS-CoV-2 showed 41 PCR-positive patients exhibited higher postoperative pulmonary complications [odds ratio (OR) 35.62; 95% confidence interval (CI) 9.34–205.55], higher thrombotic complications (OR 13.2; 95% CI 1.48—infinity), and higher 30-day mortality (OR 9.5; 95% CI 1.77–96.53) compared to matched non-COVID-19 patients [3]. A multi-center cohort study includes 235 hospitals in 24 countries and reports that out of 1128 patients who were perioperatively confirmed as PCR-positive for SARS-CoV-2, 268 (23.8%) died within 30 days [4]. Postoperative pulmonary complication occurred in 577 patients (51.2%) and of whom 219 (38.0%) died in 30 days, accounting for 81.7% of all deaths in this cohort. Most importantly, these pulmonary complications and mortality rates were the highest reported in the publications detailing postoperative outcomes in high-risk populations [4].

It is clear that being perioperative COVID-19 positive significantly affects one’s postoperative outcomes and if a situation permits, postponing or withholding surgery is recommended [3, 4]. However, we did not find the literature that reports intraoperative respiratory characteristics and ventilatory support for patients with COVID-19, and neither study described above examined the associations between intraoperative managements and those outcomes. These limited findings strongly encourage establishing a universal preoperative screening strategy to address patients who test positive with COVID-19, and further research is required to explore how intraoperative management, such as respiratory management, affects postoperative outcomes for these patients. Here we provide general background and current discussions about COVID-19 pneumonia, which may suggest recommendations of surgical management for patients who perioperatively test positive for COVID-19.

### Role of pulmonary inflammation in severe COVID-19 pneumonia

Severe COVID-19 pneumonia that deteriorated to ARDS-like lung pathology is known to be associated with elevated level of inflammatory cytokines, although the magnitude is smaller than previously reported in ARDS [5, 6]. Multiple assumptions for the pathophysiology of this disease have been made including cytokine storm, macrophage-activated syndrome (MAS) limited to the lung, type 1 and 2 interferon signals, IL-6—STAT3 axis, and neutrophil extracellular traps(NETs) [7–9]. Multiple clinical trials to antagonize the above-mentioned mediators (e.g., IL-6) are underway [7, 10, 11].

### Proposed phenotypes of COVID-19 pneumonia

Early description of a cohort of 16 COVID-19 patients in Italy by Gattinoni et al*.* proposed clinical phenotypes of patients with COVID-19 pneumonia: Type L and Type H [12, 13]. Their hypothesis is that when a patient with COVID-19 presents breathless due to pneumonia, the patient develops features of Type L first and then may progress to Type H if they do not recover. Features of Type H seem to be similar to those of severe ARDS, although it is clearly understood that ARDS is a syndrome while COVID-19 pneumonia is a disease causing the syndrome.

More precisely, Gattinoni et al*.* proposed that Type L of COVID-19 pneumonia consists of four *Low* respiratory characteristics: low elastance, low ventilation-to-perfusion, low lung weight, and low lung recruitability [12]. Elastance is the inverse of compliance so low elastance means good lung expansion. Low ventilation-to-perfusion means an increase in shunt volume due to vasoplegia, which indicates the loss of perfusion regulation and loss of hypoxic vasoconstriction, given nearly normal ventilation (i.e., gas volume) based on aerated tissue of the lung on CT scan. Considering that a CT scan of a patient in the early phase of COVID-19 pneumonia shows only ground-grass densities, low lung weight means only moderately increased lung weight. Also, a very low amount of tissue that is non-aerated leads to low lung recruitability. Another important point is that hypoxemia of Type L mainly results from vasoplegia [12]. According to a recent report, histological evaluation of autopsied lungs with COVID-19 pneumonia revealed the presence of capillary microthrombi was ninefold higher and the amount of new vessel growth though a mechanism of intussusceptive angiogenesis was 2.7-fold higher compared to the lungs from patients with H1N1 influenza [14, 15]. COVID-19 infected lungs showed distinctive vascular pathologies, including severe endothelial injury with the presence of intracellular virus and disrupted cell membranes [14]. These histological reports indicate existence of ventilation/perfusion mismatch and support the “vasoplegia hypothesis”.

When Type L patients do not improve, Gattinoni et al*.* proposed that the transition from Type L to Type H can occur. Type H consists of four high respiratory characteristics: high elastance, high right-to-left shunt, high lung weight, and high lung recruitability [12]. CT scans of Type H patients demonstrate a significantly decreased amount of aerated area due to edema, which results in high elastance (i.e., low compliance), high lung weight, and high lung recruitability, thereby increasing the fraction of cardiac output perfusing the non-aerated area (i.e., right-to-left shunt). These features of Type H fully fit the severe ARDS criteria.

Gattinoni et al*.* hypothesized how Type L COVID-19 pneumonia transitions to Type H COVID-19 pneumonia. Type L patients increase minute ventilation to respond to hypoxia due to vasoplegia, which causes a more negative intrathoracic inspiratory pressure in spontaneous breathing. Also, inflammation due to COVID-19 pneumonia increases lung permeability as observed for any type of pneumonia. The combination of these factors is known as patient self-inflicted lung injury (P-SILI) [16, 17], which may contribute to the transition process [12]. "Box 1" summarizes in the concept of P-SILI. To summarize, Type H could be treated as severe ARDS, and Type L could accept higher tidal volume of 8–9 per kg of predicted body weight with reduced positive end-expiratory pressure and early intubation may prevent the transition from Type L to Type H by limiting aggravation of P-SILI.

### Ongoing controversy of phenotypes of COVID-19 pneumonia

The proposal of clinical phenotypes of COVID-19 pneumonia has evoked strong discussion. Gattinoni hypothesized that vasoplegia may be a primary reason for Type L COVID-19 pneumonia, although Jain and Doyle argued that these different respiratory characteristics of COVID-19 pneumonia were not due to phenotypes, rather staging of pneumonia secondary to apoptosis of alveolar epithelial and endothelial cells [18]. Furthermore, Jain and Doyle argued that the early application of mechanical ventilation to patients with COVID-19 pneumonia made P-SILI unlikely to be the sole reason for disease progression.

Subsequently, cohort studies of COVID-19 pneumonia by Bos et al. and Ziehr et al. demonstrated different perspectives about the phenotypes of COVID-19 pneumonia [19, 20]. Bos et al. elucidated that there was no correlation between elastance and an estimation of lung weight in 70 patients with COVID-19 related ARDS in Amsterdam, the Netherlands, meaning low elastance of Type L does not necessarily accompany low lung weights [19]. Furthermore, most patients in their study were mixed and heterogenous in terms of the lung elastance and an estimation of lung weight. The other cohort of 66 patients with COVID-19 related ARDS in Boston, United States, were explored for their respiratory characteristics [20]. Ziehr et al*.* reported that very few patients of the cohort had near-normal compliance, which was different from the description of Type L by Gattinoni et al*.* Respiratory characteristics of the cohort described by Ziehr et al*.* were very similar to those observed in large cohorts of mechanically ventilated ARDS patients. Importantly, all patients were managed by contemporary evidence-based ARDS therapy and the mortality at 30-day follow-up was only 16.7%, although the median and interquartile range of the ratio of the partial pressure of arterial oxygen to the fraction of inspired oxygen (P/F ratio) was 182 (135–245).

Recently, Fan et al*.* summarized their viewpoint on COVID-19 related ARDS [21]. Their literature search yielded seven cohort studies of COVID-19 related ARDS, including the cohort of Gattinoni et al. Overall, the median compliance across other six studies was lower compared to the cohort observed by Gattinoni et al*.* and the number of patients in the cohort described by Gattinoni et al. was the smallest among all studies. Fan et al*.* argued that existing evidence was not enough to preclude the phenotypes of Type L with low elastance (i.e., high compliance) in patients diagnosed as having COVID-19 pneumonia; however, there has been limited evidence to initiate a different approach to patients with COVID-19 pneumonia.

### Ventilatory support for intraoperative patients who are positive with COVID-19

During this uncertain pandemic, the real problem clinicians face is to “balance the trade-off between learning (evidence of mechanism) and doing (evidence of response to treatment)” [22]. At this stage, we should keep in mind that COVID-19 patients have high mortality in the perioperative period and carefully examine the indications for surgery. Thereafter, we should continue current evidence-based approaches of intraoperative ventilatory support for COVID-19 patients without respiratory symptoms [23, 24]. As Fan et al*.* suggest, when a surgical patient presents COVID-19 related ARDS, we should focus on currently available evidence of ARDS management to treat patients with COVID-19 pneumonia given that most patients (on average) with COVID-19 related ARDS have similar respiratory characteristics of ARDS [21]. There is a chance that the novelty of clinical phenotypes of COVID-19 pneumonia may be over-weighted which leads to unintentionally ignoring risks and may result in harms to patients.

## Conclusion

The current review covers the ongoing discussion of respiratory characteristics in patients with COVID-19 and presents the limited findings from the literature that investigated intraoperative ventilatory support. We continue to explore respiratory characteristics of COVID-19 while ensuring provision of high-quality patient care. Given that preoperative PCR testing has up to 70% sensitivity and that perioperatively confirmed COVID-19 patients have a higher mortality rate, we should continue to discuss and accumulate evidence to improve perioperative patients’ outcomes. Until further evidence emerges, application of current evidence-based ARDS managements to intraoperative patients with COVID-19 related ARDS is recommended.

Box 1. Summary of Patient Self-Inflicted Lung Injury (P-SILI) The concept of P-SILI was introduced by Brochard et al*.* in 2017 to describe lung injury due to patients’ own spontaneous efforts [16]. Assisting spontaneous effort during mechanical ventilation brings various benefit such as use of less sedative agents or maintenance of diaphragm function. However, additive negative pleural pressure generated by the spontaneous effort may also be associated with exacerbation of lung injury due to increased local lung stress and overdistension [17, 25]. The focus to address these conflicting views has been (1) management of patients’ respiratory efforts, and (2) potential strategies to better protect lungs during spontaneous breathing. As such, the contemporary concept of Ventilation-Induced Lung Injury (VILI) includes lung injury induced by either a mechanical ventilation itself or patient’s own respiratory efforts (i.e., P-SILI).
